# Supplementary material for: The interactome of intact mitochondria by cross-linking mass spectrometry provides evidence for coexisting respiratory supercomplexes
Source: Mol Cell Proteomics. 2017 Dec 8;17(2):216–32. doi: 10.1074/mcp.RA117.000470 (PMC5795388; doi:10.1074/mcp.RA117.000470)
Supplement: Supplemental Data [file supp_RA117.000470_133922_0_supp_23978_fzffwf.zip › spectra_annotation/mito_spectra_annotation/82-1-8-1-9-1.pdf]

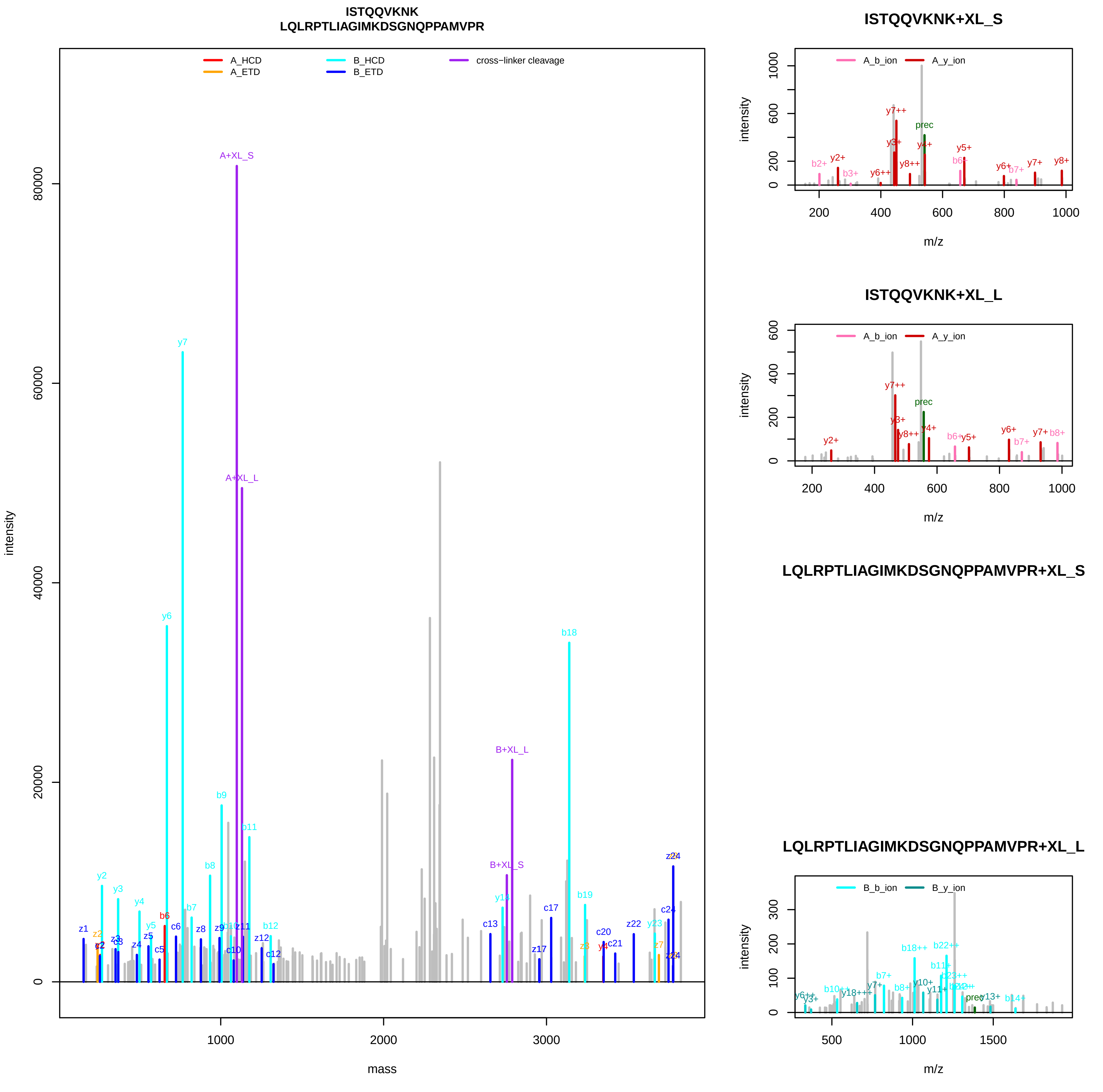
ISTQQVKNK
LQLRPTLIAGIMKDSGNQPPAMVPR
A_HCD
A_ETD
B_HCD
B_ETD
cross−linker cleavage
A+XL_S
A+XL_L
B+XL_L
B+XL_S
y7
y6
b18
b9
b11
b8
y2
y3
y4
y5
b7
b6
b12
b19
y14
y23
c13
c17
c24
z24
z1
z8
z9
z11
c10
c12
z12
z17
c20
c21
z22
intensity
mass
0
20000
40000
60000
80000
1000
2000
3000
ISTQQVKNK+XL_S
A_b_ion
A_y_ion
prec
y7++
y3+
y4+
y5+
y2+
b2+
b3+
y6++
y8++
b6+
y6+
b7+
y7+
y8+
m/z
ISTQQVKNK+XL_L
A_b_ion
A_y_ion
prec
y7++
y3+
y8++
y4+
y2+
b6+
y5+
y6+
b7+
y7+
b8+
m/z
LQLRPTLIAGIMKDSGNQPPAMVPR+XL_S
LQLRPTLIAGIMKDSGNQPPAMVPR+XL_L
B_b_ion
B_y_ion
b18++
b22++
b11+
b23++
b7+
y7+
b10++
y18+++
b8+
y10+
y11+
prec
y13+
b14+
y6++
y3+
m/z
intensity
0
100
200
300
500
1000
1500
